# Supplementary material for: Structural evidence for extracellular silica formation by diatoms
Source: Nat Commun. 2021 Jul 30;12:4639. doi: 10.1038/s41467-021-24944-6 (PMC8324917; doi:10.1038/s41467-021-24944-6)
Supplement: Supplementary file 1 — Supplementary Information [file 41467_2021_24944_MOESM1_ESM.pdf]

Supplementary Information

Structural evidence for extracellular silica formation by diatoms

Boaz Mayzel[1*], Lior Aram[1*], Neta Varsano[2], Sharon G. Wolf[3], Assaf Gal[1]

[1]Department of Plant and Environmental Sciences, Weizmann Institute of Science, Rehovot, Israel.
[2]Department of Structural Biology, Weizmann Institute of Science, Rehovot, Israel.
[3]Department of Chemical Research Support, Weizmann Institute of Science, Rehovot, Israel.
[*]These authors contributed equally to this work
Email for correspondence: assaf.gal@weizmann.ac.il

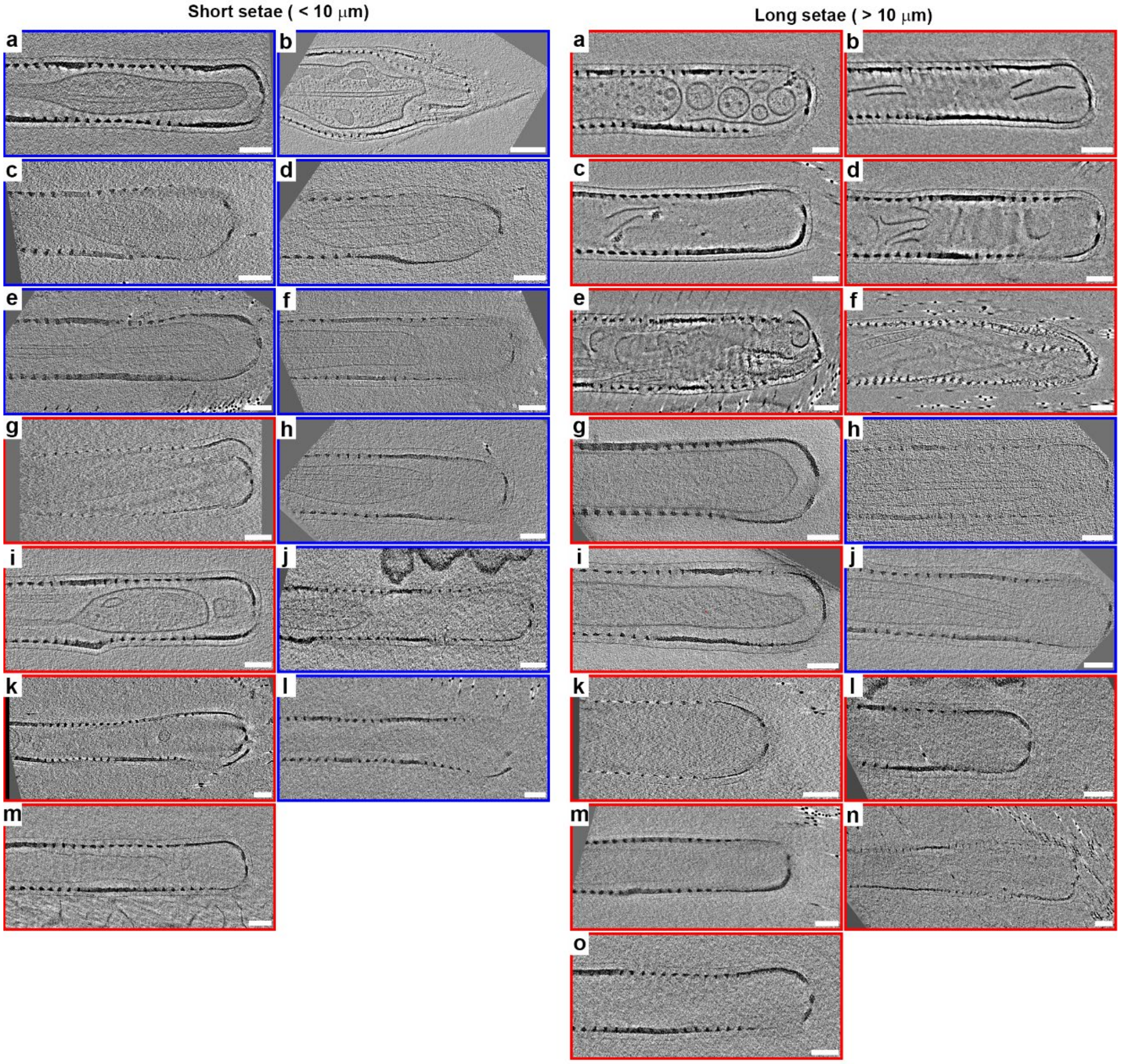

Supplementary Fig. 1. The entire cryoET datasets that are summarized in Table 1. Each panel shows a slice in the 3D reconstructed volume collected by the cryoET analyses. The setae are grouped based on their length. A panel is marked in blue if the seta has continuous cytoplasmic membrane and a complete microtubule, otherwise it is marked in red. The datasets in panels a,b from both groups appear also in the main Figures. Scale bars are 100 nm.

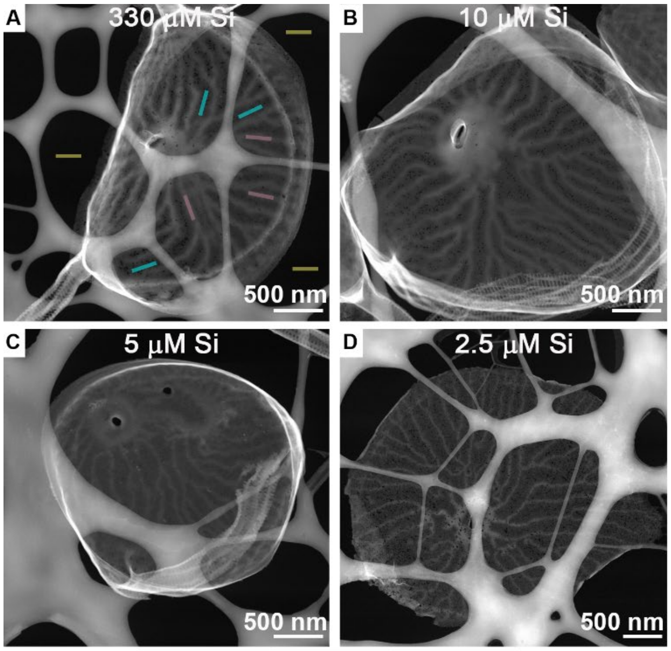

Valve region	[Si], μM	Average intensity (AU)
Costa	330	3.1±0.5
	10	4.3±0.4
	5	4.1±0.5
	2.5	2.5±0.6
Cribrum pore	330	1.6±0.4
	10	2.3±0.4
	5	2.9±0.4
	2.5	1.8±0.7

Supplementary Fig. 2. HAADF-STEM images and analyses of the Si content in the valves of *C. tenuissimus* cells grown at varying Si concentrations. A-D) images of extracted valves. The similar grey scales of the valves are indicative of similar Si content. The table provides quantification of Si content in two different regions of the valve, costa and cribrum pore. The analysis was made by averaging the background-subtracted grey values of pixels at 3 locations on the two different regions (an example is illustrated in (A) with yellow lines for background, pink for costa and light blue for cribrum pores). The constant acquisition settings allows comparing the different samples with an approximate accuracy of 10%. The table shows mean and standard deviation of 10 different valves that were imaged for each [Si]. Overall, the Si content in the valve is minimal at either very low or high Si concentrations of the growth conditions.

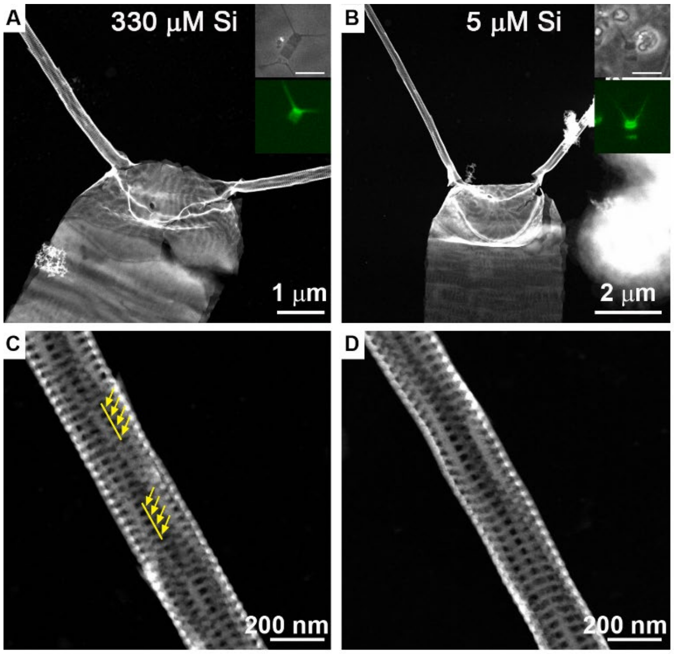

Treatment	[Si], μM	Average intensity (AU)	Number of setae
Short term	330	24±4	20
	5	27±6	20
Long term	330	28±1	13
	10	26±1	14
	5	27±1	14

Supplementary Fig. 3. HAADF-STEM images and analyses of the Si content in the cell walls of *C. tenuissimus* cultures grown at varying Si concentrations. A,B) low magnification images of newly formed cell walls, after few hours in the new medium, which were identified using correlative microscopy with PDMPO fluorescence (optical images of the same cells are in the insets, scale bars are 10 μm). C,D) High magnification images of newly formed setae. The similar grey scales of the setae are indicative of similar Si content. The table provides quantification of Si content in setae grown at different growth conditions. The analysis was made by averaging the grey values of pixels at 8 locations on the transversal elements for each seta (An example is illustrated in (C) with yellow arrows). The constant acquisition settings allow to compare the different samples with an approximate accuracy of 10%. Overall, the Si content of the setae is unchanged in all treatments.
