# Supplementary material for: Structural evidence for extracellular silica formation by diatoms
Source: Nat Commun. 2021 Jul 30;12:4639. doi: 10.1038/s41467-021-24944-6 (PMC8324917; doi:10.1038/s41467-021-24944-6)
Supplement: Supplementary file 3 — Description of Additional Supplementary Files [file 41467_2021_24944_MOESM3_ESM.pdf]

Description of Additional Supplementary Files

Supplementary Movie 1. An animation of the 3D reconstruction of the various cellular components of the seta presented in Fig. 3.
